# Real-world treatment patterns and survival for locally advanced esophageal squamous cell carcinoma

**DOI:** 10.1097/MD.0000000000034647

**Published:** 2023-08-25

**Authors:** Hua-Chun Luo, Jing-Jing Wu, Li-Jun Zhu, Lv-Juan Cai, Jing Feng, Zhi-Yong Shen, Meng-Jing Wu, Fei-Fan Chen, Zhi-Chao Fu, Fang-Wei Xie

**Affiliations:** a Department of Tumor Integrated Therapy, The Fuzhou First General Hospital Affiliated with Fujian Medical University, Fuzhou, Fujian, China; b Department of Radiotherapy, The 900th Hospital of the Joint Logistics Team, Fujian Medical University, Fuzhou, Fujian, China; c Department of Radiotherapy, Dongfang Hospital of Xiamen University, Xiamen, China.

**Keywords:** esophageal cancer, immunotherapy, locally advanced, squamous cell carcinoma, survival

## Abstract

The “real world” treatment mode and clinical efficacy of locally advanced esophageal squamous cell carcinoma (LAESCC) are unclear. Meanwhile, the role of immunotherapy in the clinical practice is also puzzling. We conducted the research to investigate the statue of “real world” LAESCC. The clinical data of patients with locally advanced esophageal squamous cell carcinoma which met the criteria from January 2010 to December 2019 have been retrospectively analyzed, and the distribution of clinical treatment patterns has been analyzed. They cover such aspects as dfferences in survival time and further analysis of the differences in overall survival (OS) and progression-free survival (PFS) between patients who received immunotherapy and those who did not receive immunotherapy. What is more, Cox risk regression model has also been used to evaluate the risk factors affecting the prognosis of LAESCC. The cases of a total of 5328 newly diagnosed patients with esophageal cancer were collected, and a total of 363 patients were included in the study, with a median age of (46.2 ± 7.8) years old; 84 (23.1%) and 279 (76.9%) patients received 1L and ≥ 2L, respectively; Concurrent chemoradiotherapy (74.1%) and paclitaxel combined with platinum-based chemotherapy (14.3%) were the main first-line treatment options; fluorouracil combined with cisplatin regimen-based chemotherapy (63.8%) was the main treatment option for ≥ 2L, of which 69 patients (25.3%) received immunization treatment; OS of patients with 1 line of therapy and ≥ 2L were (22.4 ± 7.2) months and (38.7 ± 8.5) months, respectively, and the comparison between groups was statistically significant (*P* < .05); among 69 patients with ≥ 2L who received immunotherapy, PFS and The OS was (14.6 ± 6.9) and (45.3 ± 9.7) respectively, and the comparison between the groups was statistically significant (all *P* < .05). Cox multivariate analysis has shown that clinical stage, immunotherapy, concurrent chemoradiotherapy, and ≥ 2L are the main factors affecting OS. and immunotherapy, concurrent chemoradiotherapy, and ≥ 2L are independent factors affecting PFS. Concurrent chemoradiotherapy is currently one of the standard treatments for LAESCC, and most patients are still willing to receive second-line or above treatments. Adding immunotherapy to standard treatment modalities may further optimize clinical treatment modalities and improve patient outcomes.

## 1. Introduction

Esophageal cancer is one of the most common digestive system tumors with high morbidity and mortality worldwide.^[1]^ Due to differences in region, skin color, and pathogenic factors, squamous cell carcinoma is the predominant form of esophageal cancer in the Chinese population.^[2]^ The existing diagnosis and treatment guidelines or norms are mainly based on clinical staging, and the treatment plan is selected after multidisciplinary consultation. There is no obvious difference in the treatment mode of esophageal squamous cell carcinoma or adenocarcinoma.^[3]^ Affected by the anatomical structure, about 70% of newly diagnosed esophageal cancer patients in my country have lost the indications for radical surgery when they go to the clinic, especially for patients with locally advanced esophageal cancer. How to choose a more effective treatment mode is a difficulty in current research.^[4]^ RTOG85-01 trial established the status of concurrent chemoradiotherapy for inoperable esophageal cancer. Compared with traditional radiotherapy, chemotherapy and other methods, immunotherapy, as an innovative treatment method, had its clinical efficacy confirmed in many clinical experiments,^[5]^ but as for when to intervene and how to choose the best treatment, the plan is not yet clear. After the failure of the first-line treatment, how to choose a clinical plan for second- or even third-line treatment, and whether there is a survival benefit are all blind spots in current research. This study retrospectively analyzed the clinical data of 363 patients with locally advanced esophageal squamous cell carcinoma, trying to explore the “real world” treatment mode and clinical efficacy of locally advanced esophageal squamous cell carcinoma, and further evaluate the role of immunotherapy to provide data support for clinical practice.

## 2. Materials and methods

### 2.1. General data of patients

The clinical data of patients diagnosed with esophageal cancer from January 2010 to December 2019 in the 900th Hospital of the Joint Logistics Support Force of the Chinese People’s Liberation Army were retrospectively analyzed. Inclusion criteria: The diagnosis of esophageal squamous cell carcinoma was confirmed by histopathological examination; Bone whole body imaging, upper abdominal MRI, abdominal color Doppler ultrasound or PET-CT examination, no distant organ metastasis; Through chest CT and gastric Intestinal barium meal examination, no indication of radical surgery after multidisciplinary consultation; Eastern cooperative oncology group score of 0 to 2; Age ≤ 75 years old; Complete clinical data. Exclusion criteria: Patients with severe and uncontrollable underlying diseases; Patients with a history of malignant tumors; Patients who are unwilling to receive long-term follow-up; Patients with psychiatric diseases; Incomplete clinical data. This study was approved by the hospital ethics committee, and all patients signed informed consent.

### 2.2. Determination of treatment mode

First-line treatment is defined as the treatment mode that patients receive after diagnosis; second-line treatment is defined as a new treatment mode after patients receiving first-line treatment due to disease progression or intolerance of the side effects of first-line treatment. The time interval is >21 days; the third-line treatment is defined as receiving a new treatment mode after receiving the second-line treatment due to disease progression or intolerance of the side effects of the first-line treatment, and the time interval between the second-line treatment and the second-line treatment is >21 days. All treatment modes can be single or single drug treatment methods, and are not limited to 2 or more drugs or treatment methods.

### 2.3. Treatments

#### 2.3.1. Chemotherapy alone paclitaxel combined with cisplatin.

Paclitaxel 135 mg/m2 intravenous drip d1, cisplatin 75 mg/m2 intravenous drip d1, q3w; fluorouracil combined with cisplatin regimen (PF): 750 mg/m2 CIV d1-5, cisplatin 75 mg/m2 intravenous drip d1, q3w.

#### 2.3.2. The concurrent radio chemotherapy and radiotherapy plan is as follows.

Thermoplastic body membrane combined with vacuum pad for body position fixation, the patient is placed in the supine position, hands placed in front of the forehead, Philips large aperture 4.0 CT scanning is positioned, and the scanning range is from the level of the hyoid bone to the lower border of the right kidney; the slice thickness is 5 mm, and the scanned image is transmitted to the Eclips treatment planning system. Target area delineation principle: delineate the target area in combination with baseline evaluation of gastrointestinal barium meal examination, chest CT and electronic gastroscopy. GTV is the esophageal tumor lesion, which is 1 cm in the upper and lower directions, and 0.6 cm in the other directions as PGTV. The dose of 95% PGTV is 1.8 to 2.0 Gy/time, 1 time/day, 5 times/week, a total of 25 to 30 times, and the total amount is 50 to 60 Gy; GTVnd is The enlarged lymph nodes can be seen on CT, and the PGTVnd is 0.5 cm from each side. The dose of 95% PGTVnd is 1.8 to 2.0 Gy/time, 1 time/day, 5 times/week, a total of 25 to 30 times, and the total amount is 50 to 60Gy; CTV is the mediastinal prevention lymph node. The drainage area varies according to the location of the primary tumor in the esophagus. The lymph node drainage area of upper thoracic esophageal cancer includes supraclavicular lymph node drainage area, paraesophageal lymph node drainage area, group 2, group 4, group 5, and group 7 lymph node drainage area; lymph node drainage area of middle thoracic esophageal cancer includes paraesophageal lymph node drainage area, 7 groups of lymph node drainage areas; the lower thoracic lymph node drainage areas include paraesophageal, 4, 5, 7, and left gastric and paracardial lymph node drainage areas; CTV externally 0.3 cm is PCTV, and the dose of 95% PCTV is 1.8 to 2Gy/ times, 1 time/day, 5 times/week, a total of 30 times, with a total amount of 50 to 54Gy. Normal organ dose limits are as follows: V20 ≤ 20% for both lungs, V45 ≤ 0% for spinal cord, and V30 ≤ 5% for heart.

#### 2.3.3. Concurrent chemotherapy regimen.

Cisplatin 30 mg/m2 intravenous infusion, d1 to 2 combined with albumin paclitaxel injection 260 mg/m2 intravenous infusion, d1, a total of 2 cycles of concurrent chemotherapy during the concurrent chemoradiotherapy.

#### 2.3.4. Immunotherapy.

ICI includes pembrolizumab, toripalimab, camrelizumab, sintilizumab, and tilelizumab. The doses of pembrolizumab, carrelizumab, sintilizumab, and tilelizumab received by the patients were fixed doses of 200 mg every 3 weeks. The therapeutic dose of toripalimab is a fixed dose of 240 mg every 3 weeks. All patients included in the study received at least 1 cycle of the above immunization regimen until tumor progression.

### 2.4. Observation indicators

Tumor staging was based on the 2017 TNM staging standard of the American Joint Committee on Cancer. To compare the difference in general information and tumor objective response rate (ORR) between only one line of therapy (IL) and at least 2 lines of therapy, Among them, ORR = [number of complete response (CR) + number of partial response (PR)]/total number*100%; overall survival (OS) between patients with IL and ≥ 2L; difference in OS and progression-free survival (PFS) between patients who received immunotherapy and those who did not receive immunotherapy; and further The prognostic factors affecting OS were analyzed.

The overall survival time was defined as the time from the day of diagnosis to death or loss to follow-up; the time of disease progression-free survival was defined as the time from the day of diagnosis to the imaging-proven tumor progression (PD) and/or the appearance of new metastatic lesions. The efficacy evaluation criteria were based on the WHO RECISIT criteria. CR was defined as the complete disappearance of esophageal lesions and the disappearance or reduction of metastatic lymph nodes to normal (short diameter < 1 cm); PR was defined as the sum of the diameters of esophageal lesions and mediastinal lymph node lesions. Shrinkage ≥ 30%; PD is defined as a ≥ 20% increase in the sum of diameters of esophageal and mediastinal lymph node lesions compared to baseline assessment or the appearance of any 1 new lesion; esophageal and mediastinal lymph node lesions in the SD definition, The criterion for the sum of diameters is between PR and PD. The CR, PR, PD, and SD were finally confirmed through the joint confirmation of an associate chief imaging physician and associate chief clinical physician.

### 2.5. Follow-up

The follow-up time was up to December 31, 2020, and the median follow-up time was (42.4 ± 7.8) months. Follow-up methods included telephone, inpatient and outpatient follow-up. Review every 3 months for the first 2 years after treatment, and every 6 months for the 3rd to 5th years. Review items include chest CT, abdominal color Doppler ultrasound, gastrointestinal barium meal, blood routine, liver and kidney function, etc.

During the follow-up process of all patients, if the disease progresses, the treatment plan needs to be changed. That is, the increase in the number of treatment lines needs to be confirmed by 2 deputy chief physicians; if the 2 deputy chief physicians disagree, the third chief physician will carry out Decision; if the time of disease progression is ≥ 6 months from the last treatment, the chemotherapy regimen remains unchanged, and it is still included in the category of ≥ 2L.

### 2.6. Statistical methods

Statistical analysis was performed using SPSS software (version 26.0, IBM Software, Armonk, NY). Descriptive test was used to count the general data of patients with IL and ≥ 2L, and chi-square test or t test was used for comparison between groups; Square test; OS and PFS analysis using Kaplan–Meier method, parallel Log-rank test; Cox hazard regression model was used to evaluate the prognostic risk factors affecting OS in patients with esophageal cancer. *P* < .05 was considered to be statistically significant.

## 3. Results

### 3.1. General information of research subjects

**Figure 1.**
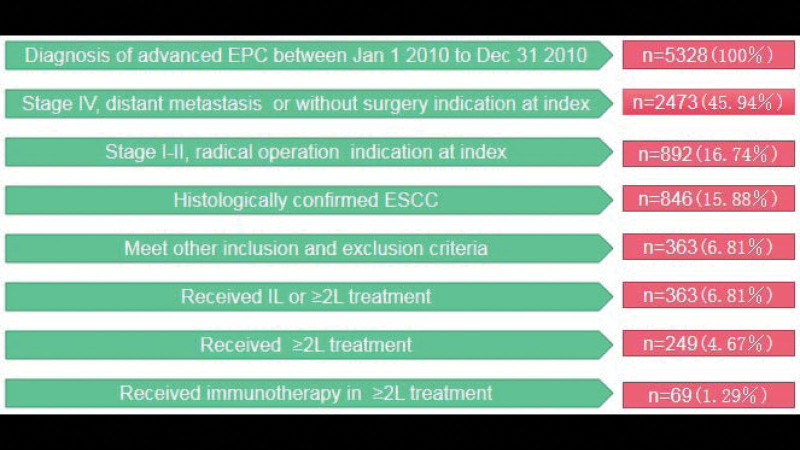
All patients screening flow chart (esophage cancer, EPC; esophageal squamous cell carcinoma, ESCC).

Casees of a total of 5328 newly diagnosed patients with esophageal cancer were collected, and a total of 363 patients met the inclusion and exclusion criteria, 136 (37.5%) females, 84 (23.1%) patients received IL therapy only, 279 (76.9%) patients received ≥ 2L regimen treatment, and only 69 patients received immunotherapy in ≥ 2L regimen treatment, See Figure 1.

**Figure 2.**
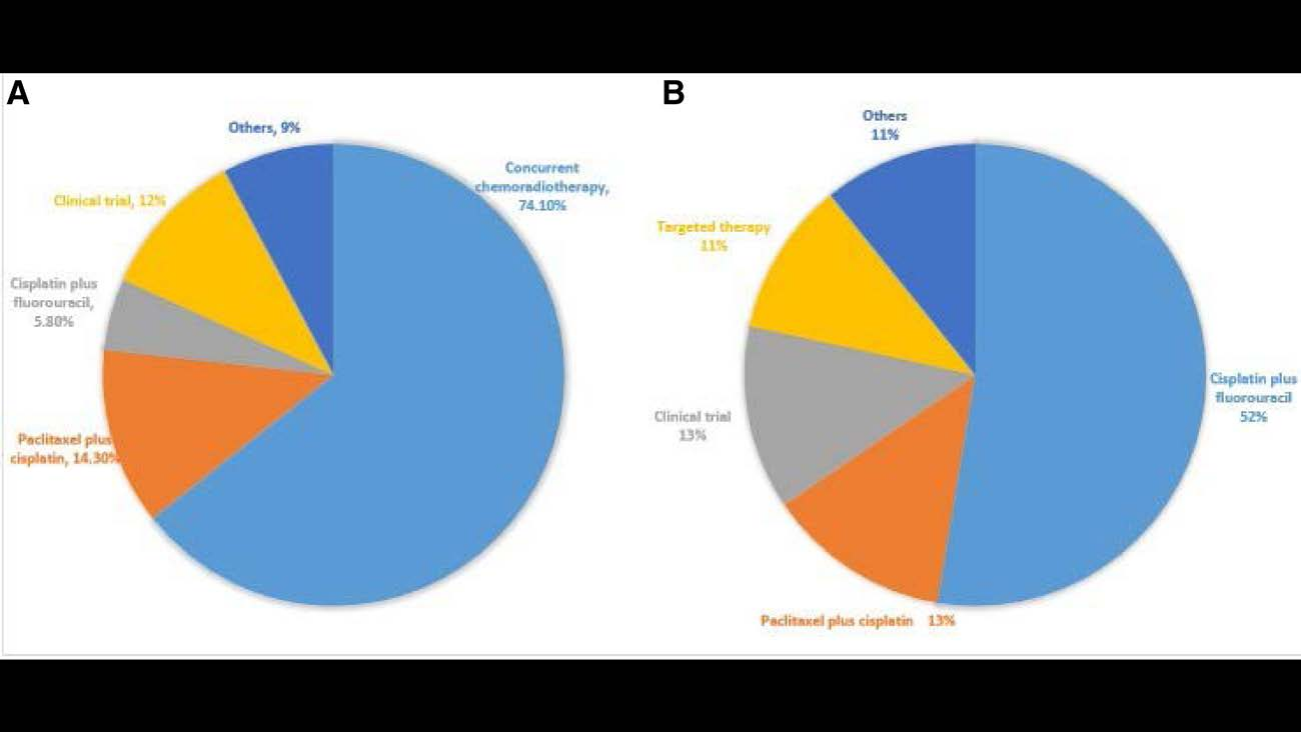
(A) Breakdown of treatments received in the first-line setting, (B) breakdown of treatments received in the ≥ 2-line setting.

Among the first-line treatment regimens, the largest proportion was concurrent chemoradiotherapy in 269 cases (74.1%), followed by paclitaxel combined with platinum-based chemotherapy in 52 cases (14.3%); among ≥ 2L regimens, the largest proportion was PF-based chemotherapy regimen 147 cases (52.7%), followed by paclitaxel combined with platinum-based chemotherapy in 36 cases (12.9%), and 36 patients (12.9%) participating in clinical trials. In the ≥ 2L regimen, 69 patients (25.3%) received immunotherapy. See Figure 2.

### 3.2. Comparison of general conditions of patients in IL group and patients in ≥ 2L group.

The general conditions of the 2 groups of patients, such as age, gender, Eastern cooperative oncology group score, TNM stage, tumor location, length were comparable (*P* > .05). After first-line treatment, the ORR of the 2 groups of patients was comparable. The rates were 90.5% and 87.8%, respectively, and there was no statistical difference between the groups (*P* > .05). See Table 1.

**Table 1:** Baseline characteristics of patients with advanced esophacacinoma cancer who received at least one line of systemic therapy (n, %).

Date	IL (n = 84)	≥2L (n = 279)	χ^2^ value	*P* value
Age			0.28	.59
≥65	21 (25.0%)	62 (22.2%)		
<65	63 (75.0%)	217 (77.8%)		
Sex			2.58	.11
Man	37 (44.0%)	96 (34.4%)		
Female	47 (56.0%)	183 (65.6%)		
ECOG scores			0.02	.98
0	23 (27.4%)	79 (28.3%)		
1	22 (26.2%)	72 (25.8%)		
2	39 (46.4%)	128 (45.9%)		
TNM stage (baseline)			0.31	.57
III	35 (41.7%)	126 (45.2%)		
IVa	49 (58.3%)	153 (54.8%)		
Curative effect			0.78	.53
CR	5 (6.0%)	21 (7.5%)		
PR	71 (84.5%)	224 (80.3%)		
SD	7 (8.3%)	29 (10.4%)		
PD	1 (1.2%)	5 (1.8%)		
Tumor site			0.41	.93
Cervical segment	7 (8.3%)	26 (9.3%)		
Upper thoracic	12 (14.3%)	45 (16.1%)		
Middle thoracic	34 (40.5%)	114 (40.9%)		
Lower thoracic	31 (36.9%)	94 (33.7%)		
Degree of pathological differentiation			0.36	.83
Highly	46 (54.8%)	158 (56.6%)		
Moderately	25 (29.8%)	74 (26.6%)		
Poorly	13 (14.4%)	47 (16.8%)		
Length of tumor			1.3	.71
≤3 cm	3 (3.6%)	12 (4.3%)		
> cm and <5 cm	32 (38.1%)	111 (39.8%)		
≥5 cm and <7cm	34 (40.5%)	120 (43.0%)		
≥7cm	15 (17.8%)	36 (12.9%)		

≥2L = at least two lines of therapy, CR = complete response, ECOG = Eastern cooperative oncology group, IL = one line of therapy, PR = partial response.

### 3.3. Comparison of OS between IL and ≥ 2L patients

**Figure 3.**
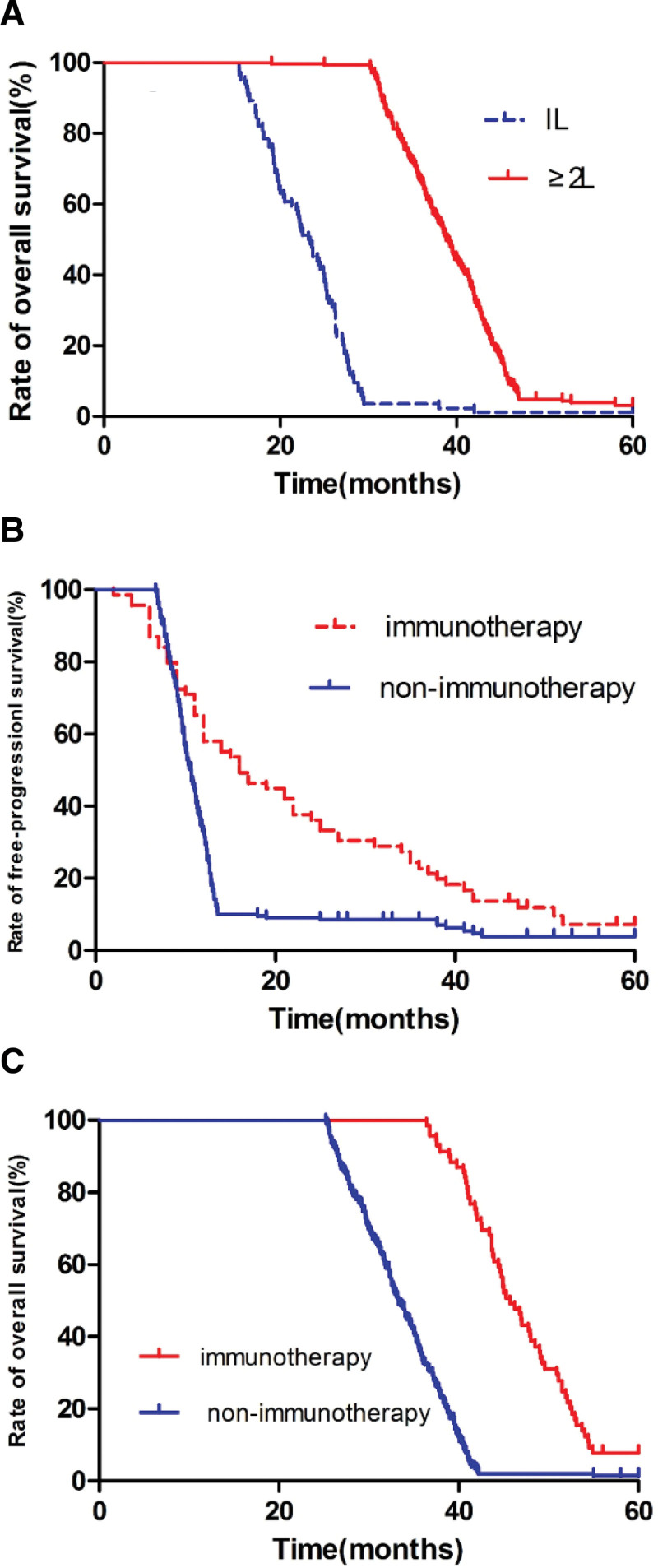
(A) Overall survival curve between IL and ≥ 2L, (B) free-progression survival between immunotherapy and nonimmunotherapy, and (C) overall survival curve between immunotherapy and nonimmunotherapy. IL = one line of therapy.

The OS of IL patients was (22.4 ± 7.2) months, and the OS of ≥ 2L patients was (38.7 ± 8.5) months, and there was a statistically significant difference between the groups (2 = 322.9, *P* < .05)., (95% CI: −0.18 to 1.38). See Figure 3.

### 3.4. Comparison of PFS and OS with and nonimmunotherapy

Among them, 279 patients with ≥ 2L, 69 (24.7%) received immunotherapy, PFS and OS were (14.6 ± 6.9) months and (45.3 ± 9.7) months, respectively, 210 patients (75.3%) who did not receive immunotherapy, PFS and OS were (10.2 ± 3.5) months and (33.8 ± 8.6) months, respectively, and there were significant differences in PFS and OS between those who received and those who did not receive immunotherapy (*P* < .05). See Figure 3.

### 3.5. Multivariate analysis affecting the prognosis of patients with locally advanced esophageal cancer

The univariate analysis showed the patients who received immunotherapy, concurrent chemoradiotherapy and ≥ 2L therapy had better prognosis. The result was the same for the patients whose TNM stage was III stage. See Table 2. The clinical stage, immunotherapy, concurrent chemoradiotherapy, and ≥ 2L were included in the Cox proportional hazards regression model. Independent factors; while immunotherapy, concurrent chemoradiotherapy, and ≥ 2L were independent factors affecting PFS. See Table 3.

**Table 2:** The univariate analysis of prognostic factors for locally advanced esophageal cancer (%).

Date	NO.	1 year	3 years	5 years	χ2 value	*P* value
Age					0.456	.500
≥65	83 (25.0)	81 (97.6)	37 (44.6)	19 (22.9)		
<65	280 (75.0)	278 (99.3)	108 (38.6)	57 (20.3)		
Sex					0.083	.073
Man	133 (44.0)	132 (99.2)	49 (36.8)	24 (18.0)		
Female	230 (56.0)	227 (98.7)	96 (41.7)	52 (22.6)		
TNM stage (baseline)					6.369	.000
III	161 (41.7)	161 (100.0)	83 (51.6)	49 (30.4)		
IVa	202 (58.3)	198 (98.0)	62 (30.7)	27 (13.4)		
Immunotherapy					9.117	.003
Yes	69	69 (100.0)	39 (56.5)	22 (31.9)		
No	294	290 (98.6)	106 (36.1)	54 (18.4)		
Concurrent chemoradiotherapy					7.291	.001
Yes	269	269 (100.0)	106 (39.4)	61 (22.7)		
No	94	90 (95.7)	39 (38.3)	15 (16.0)		
Lines					9.473	.002
≥2L	279	278 (99.6)	111 (39.8)	57 (20.4)		
1L	84	81 (96.4)	34 (40.5)	19 (22.6)		

≥2L = at least two lines of therapy.

**Table 3:** Cox multivariate analysis of influencing prognosis of local advanced esophageal cancer.

Variable	OS			PFS		
	B	HR (95% CI)	*P* value	B	HR (95% CI)	*P* value
TNM (IVa vs III)	3.24	23.27 (4.76–98.02)	<.01	-	-	-
Immunotherapy (no vs yes)	1.69	4.00 (1.78–18.69)	<.01	1.63	5.71 (2.22–13.47)	<.01
Concurrent chemoradiotherapy (no vs yes)	1.27	3.72 (1.91–8.62)	<.01	2.14	8.39 (2.98–23.88)	<.01
Lines (1L vs ≥2L)	1.46	4.58 (2.37–11.79)	<.01	3.38	7.64 (3.91–16.69)	<.01

≥2L = at least two lines of therapy, OS = overall survival, PFS = progression-free survival.

## 4. Discussion

Esophageal cancer cells proliferate actively and have poor sensitivity to radiotherapy and chemotherapy. Locally advanced esophageal cancer has a large tumor burden and a high risk of recurrence, metastasis, esophageal perforation, bleeding, and other complications.^[6]^ The development of radiotherapy technology, the optimization of chemotherapy drugs, targeted drugs, anti-vascular drugs and other treatment methods, especially the emergence of immunotherapy in many clinical trials of esophageal cancer, have changed the traditional treatment mode of locally advanced esophageal cancer. Whether patients with locally advanced esophageal cancer can receive the best regimen in the “real world” treatment, and whether immunotherapy can improve the prognosis of patients with esophageal cancer after failure of first-line therapy, remain controversial.

Among the 5328 patients with esophageal squamous cell carcinoma in the past 10 years, there were 363 patients with locally advanced unresectable patients, accounting for 6.8%. The occurrence of esophageal squamous cell carcinoma is related to smoking, drinking, poor dietary habits, genetic factors, and lack of micronutrients. Due to the lack of screening guidelines for esophageal squamous cell carcinoma, patients may delay the best time for treatment.^[7]^ Although the development of imaging technology has improved the diagnosis rate of esophageal squamous cell carcinoma, if the screening of early gastroscope is actively carried out, the diagnosis rate of early esophageal squamous cell carcinoma can be effectively improved.^[8]^ Whether the proportion in the total population shows a decreasing trend year by year needs further research. In European and American countries, the incidence of esophageal squamous cell carcinoma has shown a downward trend, but the incidence of males has increased to varying degrees.^[9]^ In this study, the proportion of male patients was significantly higher than that of female patients (62.5% vs 37.5%), which was significantly related to regional ethnic distribution and incidence factors. The age group of 40 to 60 years is the high incidence age of esophageal cancer, but the clinical treatment costs vary greatly depending on the age of onset and histological stage.^[10,11]^ The median age of locally advanced esophageal squamous cell carcinoma in this study was (47.4 ± 6.8) years, which was basically consistent with previous reports. Among people over 40 years old, popularizing the early screening of electronic gastroscope can improve the detection rate of early esophageal squamous cell carcinoma and minimize the expenditure of medical insurance.

Concurrent chemoradiotherapy is one of the standard treatment options for patients with locally advanced esophageal cancer, but the overall effect is poor. The treatment mode of different chemotherapy regimens combined with radiotherapy has gradually become a research hotspot.^[12]^ The modality of concurrent radiotherapy with paclitaxel, cisplatin, and fluorouracil (DCF-RT) has a better prognosis than concurrent radiotherapy with cisplatin and fluorouracil (CF-RT), and there is no significant increase in clinical adverse effects.^[13]^ In the real-world, nearly 269 patients (74.1%) received concurrent radiotherapy with paclitaxel combined with cisplatin, which also indirectly reflects the concurrent radio chemotherapy regimen of 2 or more drugs, which is the first choice for first-line treatment. It can inhibit the proliferation of tumor cells to the greatest extent and achieve the purpose of improving the efficacy,^[14]^ but there is no consensus on the optimal concurrent chemoradiotherapy regimen. With the deepening of immunotherapy research, in many large clinical studies, the addition of immunotherapy has significantly improved the prognosis compared with chemotherapy alone.^[15,16]^ However, affected by my country’s economic factors and medical insurance system, chemotherapy alone in the first-line regimen is mainly paclitaxel combined with platinum (14.3%). With the approval of immunotherapy drugs for esophageal squamous cell carcinoma indications, the medical treatment strategy for patients with unresectable locally advanced esophageal squamous cell carcinoma will also be completely changed. Radiation therapy has the effect of destroying the DNA double-strand of tumor cells, improving antigen exposure and presentation, and has a synergistic effect with immunotherapy.^[17]^ Adding immunotherapy on the basis of the 3-drug combination can theoretically overcome the limitation of cross-resistance and improve the control rate of esophageal lesions, but there is no prospective study to confirm the side effects and safety. Therefore, in the first-line treatment regimen, concurrent chemoradiotherapy is still the current mainstream mode, and concurrent chemoradiotherapy with chemotherapy drugs combined with immunotherapy may be the best choice.

Studies have confirmed that although the palliative treatment mode can reduce the side effects of treatment to a certain extent, it does not improve the overall survival rate. For patients with esophageal squamous cell carcinoma who have received multiple lines of treatment, more attention should be paid to comprehensive treatment based on nutritional support.^[18]^ In the real-world, esophageal squamous cell carcinoma has a high risk of recurrence or metastasis after first-line therapy due to its poor sensitivity to chemoradiotherapy.^[19]^ However, most of the patients did not have advanced tumor manifestations such as cachexia, and the overall physical condition was still in the stage of tolerable treatment. Therefore, 279 patients (76.9%) received ≥ 2L regimen. There were 147 cases (52.7%) who received PF-based chemotherapy regimen. After first-line paclitaxel combined with platinum regimen, PF was used as a continuous chemotherapy strategy in second-line regimen, which indirectly confirmed that paclitaxel, cisplatin, and fluorouracil are the main consolidation of esophageal squamous cell carcinoma. Chemotherapy, either at baseline or second-line.^[20]^ In a multi-center study program, the above 3 chemotherapy drugs were used as the research objects, and they were combined with each other. In the progress of the initial diagnosis program, other programs were used as continuous treatment.^[21]^ This study is basically consistent with this treatment model. As a necessary stage of drug research and development, clinical trials have gradually put the interests of patients in the most important position, and ensured the safety and efficacy of patients through blind review, supervision and other measures.^[22]^ Thirty-six patients (12.9%) in the ≥ 2L regimen participated in the clinical trial. Compared with the treatment mode of IL, there was a greater improvement, mainly because there was no effective second-line treatment regimen for esophageal cancer. The research sponsor urgently needs This situation is changed through clinical trials; it also indirectly shows that the concept of clinical trials is more and more accepted by the Chinese community. Currently, the approved immunotherapy drugs for esophageal cancer are mainly programmed death-1 and programmed death-ligand 1 (PD-L1). In 2017, the US Food and Drug Administration approved pembrolizumab for the treatment of recurrent localized tumors that express PD-L1 (CPS ≥ 1) and progress after receiving 2 or more chemotherapy regimens After advanced gastroesophageal junction adenocarcinoma; pembrolizumab was approved in 2019 for recurrent, locally advanced, or metastatic esophageal cancer after first- or multiple-line systemic therapy. In recent years, domestic drugs such as carelizumab and sintilimab have gradually been written into the first-line treatment guidelines from the third-line. Limited by objective factors such as study time, in the past 10 years, although only 69 patients (25.3%) received immunotherapy, and all of them were used after first-line treatment, this also indirectly reflects the rapid progress of drug research and development in China in recent years. the China Food and Drug Administration has also accelerated the approval process for clinical oncology drugs.

After further stratified analysis, the overall survival time of patients receiving ≥ 2L regimen was significantly better than that of IL patients, and clinical stage, immunotherapy, concurrent chemoradiotherapy, at least 2 lines of therapy and other factors were closely related to prognosis. For patients with esophageal squamous cell carcinoma, consistent with other solid tumors, the tumor burden is positively related to the prognosis of the patients, and the local control rate of the tumor should be emphasized.^[23]^ The OS of patients with IL and ≥ 2L in this study were (22.4 ± 7.2) months and (38.7 ± 8.5) months, respectively, which were significantly higher than the 13.1 months reported by Janjigian,^[24]^ who only used chemotherapy combined with immunotherapy, confirming that Radiation therapy plays a very important role in locally advanced unresectable esophageal squamous cell carcinoma. Differences in pathology types and study populations may cause bias in study results. Immunotherapy significantly improved PFS and OS in patients with ≥ 2L, which was consistent with the findings of Zhang et al,^[25]^ further confirming that immunotherapy may have synergistic effects with radiotherapy and chemotherapy.^[26]^ After concurrent chemoradiotherapy, neutrophils were significantly increased, and tumor mutation load and neoantigen load were significantly reduced, which may be the mechanism of action of potential immunotherapy.^[27]^

However, limited by the actual sample size included, our conclusions may be biased. Due to the large deviation in sample size at each stage, there would be bias in the research results. In addition, the choice of different immunological drugs and combination regimens may affect the final survival data. Due to the retrospective design, we did not pay attention to the expression of esophageal cancer-related molecules such as HER-2 and PD-L1 in patients. Furthermore, in real-world studies, we may not be able to focus on some concomitant diseases in which patients show no symptoms other than the tumor.

## 5. Conclusion

In the era of immunotherapy, the prognosis of unresectable locally advanced esophageal squamous cell carcinoma is expected to be further improved. In China’s unique medical environment, concurrent chemoradiotherapy as a standard treatment mode is being more closely integrated with immunotherapy. According to the biological characteristics of esophageal squamous cell carcinoma in China, the development of effective and reasonable first- and second-line treatment strategies can further improve the survival time of patients.

## Author contributions

**Conceptualization:** Huachun Luo, Fang-Wei Xie.

**Data curation:** Jing-Jing Wu, Jing Feng, Meng-Jing Wu.

**Formal analysis:** Zhi-Chao Fu, Fang-Wei Xie.

**Funding acquisition:** Huachun Luo.

**Investigation:** Li-Jun Zhu, Fei-Fan Chen.

**Methodology:** Lv-Juan Cai, Zhi-Yong Shen.

**Writing – original draft:** Huachun Luo.
